# Supplementary material for: The Assessment and the Within-Plant Variation of the Morpho-Physiological Traits and VOCs Profile in Endemic and Rare Salvia ceratophylloides Ard. (Lamiaceae)
Source: Plants (Basel). 2021 Mar 3;10(3):474. doi: 10.3390/plants10030474 (PMC7998927; doi:10.3390/plants10030474)
Supplement: Supplementary file 1 [file plants-10-00474-s001.pdf]

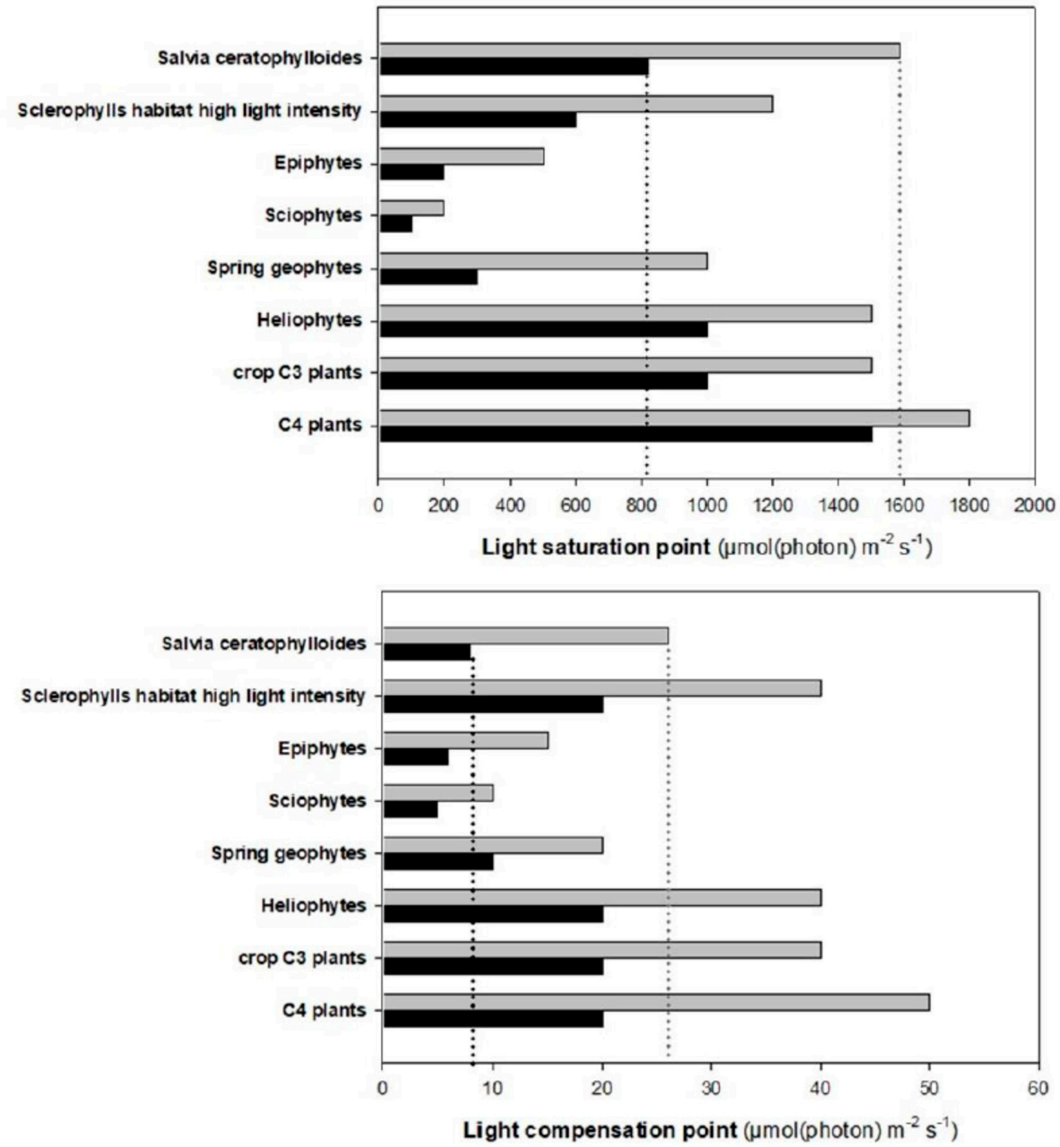

Figure S1. Light saturation point (μmol(photons) m^{-2} s^{-1}) (upper panel) and light compensation point (μmol(photons) m^{-2} s^{-1}) (bottom panel) of different plant functional groups. The data [minimum (■) and maximum value (□)] are derived from Larcher [80]. The dotted lines are drawn for a better comparison with the minimum and maximum value of *Salvia ceratophylloides*.

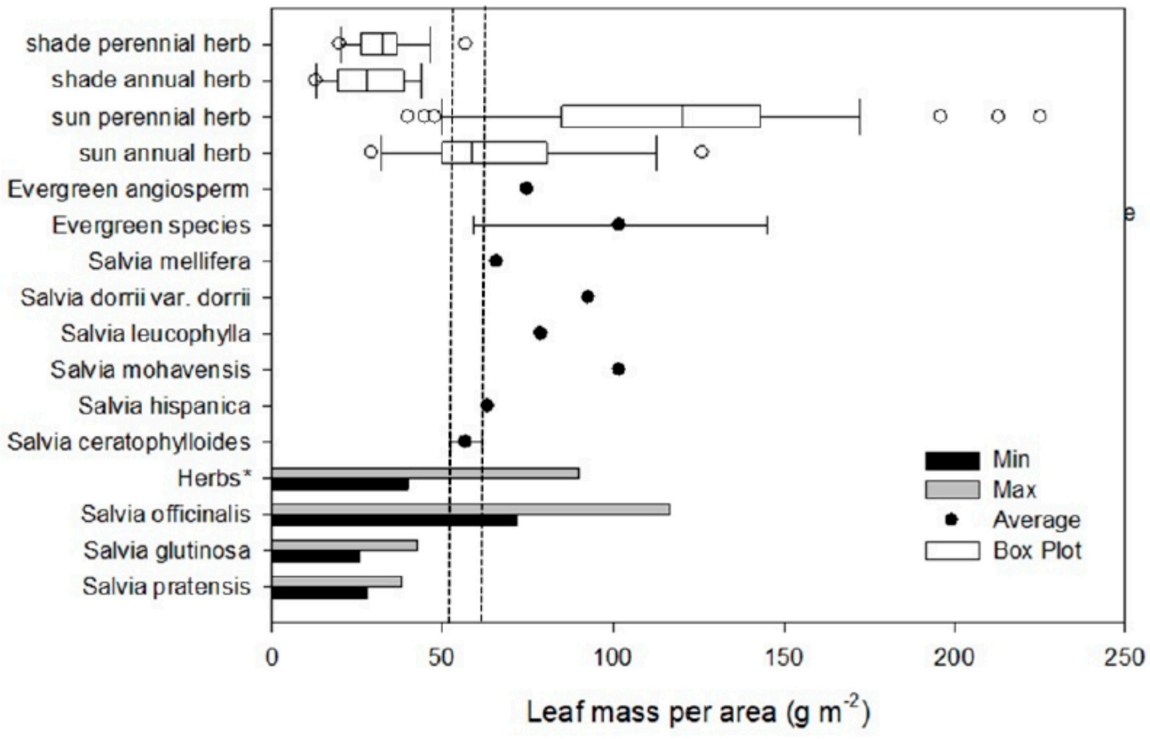

Figure S2. Leaf mass per area (g m-2) of sun- and shade-species herbs, evergreen angiosperm and species, herbs and different *Salvia* species. The data of LMA of Salvia species, herbs, evergreen angiosperm and species are indicated by minimum (■) and maximum value (■) or by the average (black plot point and the standard deviation where reported)] and have been derived from Martins et al. [82] for *S. officinalis*, Mommer et al. [34] for S. pratensis, Paz ́-Dyderska et al. [35] for *S. glutinosa*, Goergen et al. [33] for *S. hispanica*, Knight and Ackerley [31] for *S. mohavensis*, *S. leucophylla*, *S. dorrii* var. dorrii and *S. mellifera*, Poorter et al. [29] for herbs, Duursma et al. [81] for evergreen angiosperm and de la Riva et al. [30] for evergreen species. Box plots point out the distribution of LMA values as observed for a wide range of sun- and shade-species herbs both annual and perennial, with the bottom and top part of the box indicating the 25th and 75th percentile, respectively, the two whiskers the 10th and the 90th percentile, respectively, and the horizontal line within the box the median value. The data for the box plot are derived by scientific literature as indicated in Table S1. The dotted lines have been drawn for better comparisons and pointed out the range of LMA values of *S. ceratophylloides*.

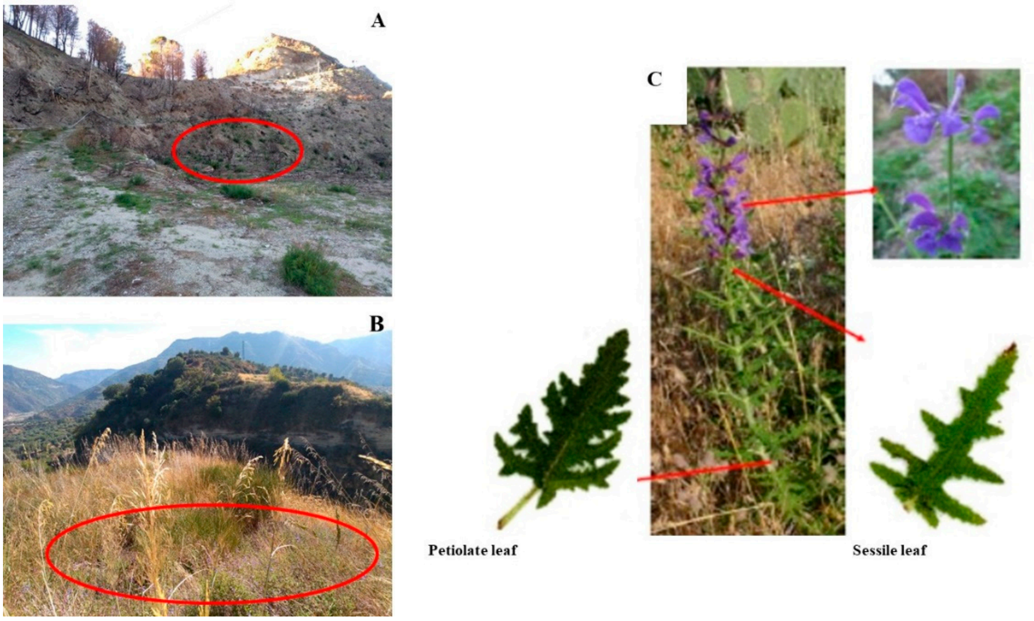

Figure S3. - Plants of *Salvia ceratophylloides* in the two sites, Mosorrofa [Mo] (A) and Puzzi [Pu] (B). The red circles indicate the places where the *Salvia ceratophylloides* plants have been discovered. (C) Individual plant of *Salvia ceratophylloides* and different leaves: petiolate (P) and sessile leaf (S)

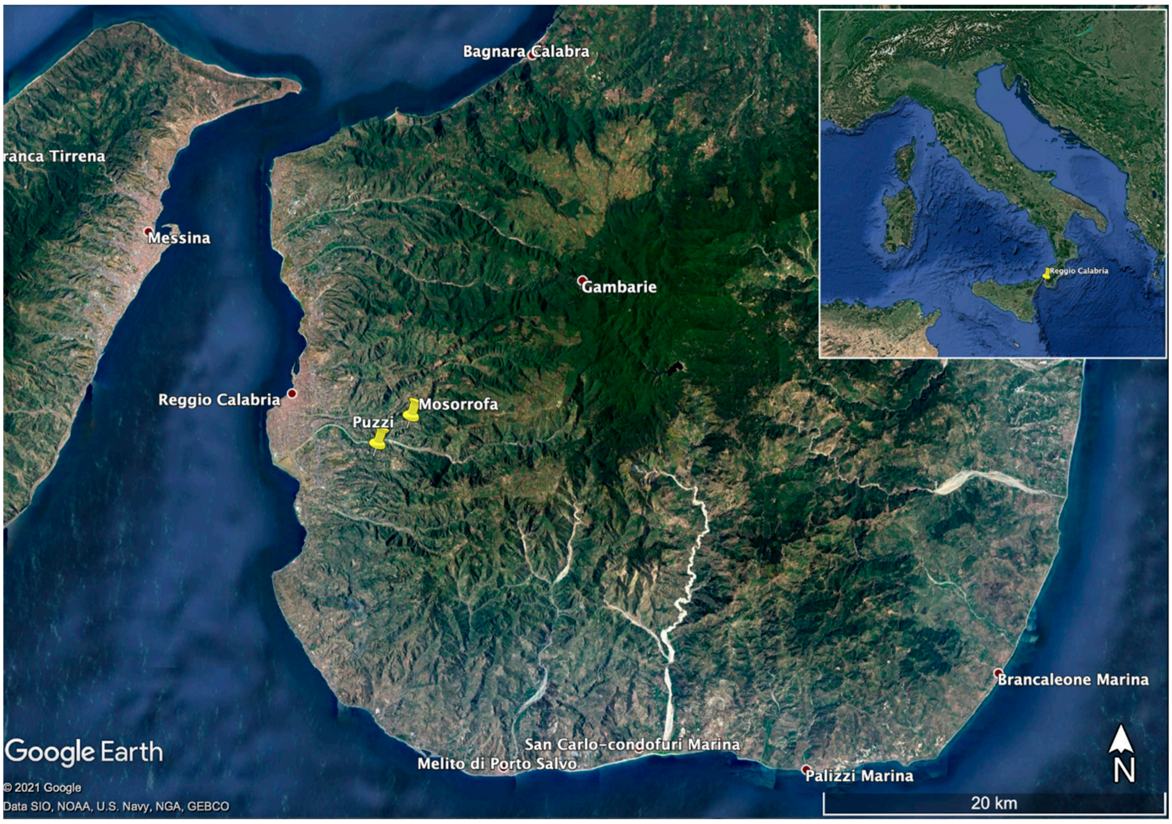

Figure S4. - Distribution map of *Salvia ceratophylloides*.

Table S1 – Two-way ANOVA results and chemical characterization (average and error standard within brackets) of volatile organic compounds in fresh sessile and petiolate leaves of *Salvia ceratophylloides* harvested in two different sites [Mosorrofa (Mo) and Puzzi, (Pu)]. Different lower-case letters indicated significant differences at $p < 0.05$ among the average along the rows (Tukey' test) and they have been only reported when the LT × Sit interaction was significant. The bold identify the statistically significant factors and/or their interaction.

	Compound	Chemical classes	#Statistics	Sessile		Petiolate	
				Pu	Mo	Pu	Mo
1	Camphene	Monoterpene	LT 0.75^{NS}<br>Sit 0.64^{NS}<br>LT × Sit 1.25^{NS}	468	141	130	184
2	Camphor		LT 2.78^{NS}<br>Sit 4.82^{NS}<br>LT × Sit 0.86^{NS}	1776	368	606	34
3	Limonene		LT 5.30^{NS}<br>Sit 1.33^{NS}<br>LT × Sit 0.74^{NS}	77187	72261	53128	19515
4	p-Cymene		**LT 7.78***<br>**Sit 14.16****<br>LT × Sit 0.21^{NS}	195813	82392	108569	19607
5	Pinocarvone		LT 0.04^{NS}<br>**Sit 6.57***<br>LT × Sit 0.07^{NS}	2406	987	2444	696
6	Sabinene		**LT 11.80****<br>Sit 0.34^{NS}<br>LT × Sit 1.70^{NS}	554775	873306	195242	73210
7	Terpinolene		**LT 12.40****<br>Sit 0.40^{NS}<br>LT × Sit 3.09^{NS}	128554	218320	62198	19784
8	trans-Sabinene hydrate		LT 0.00^{NS}<br>Sit 0.28^{NS}<br>LT × Sit 3.73^{NS}	3086	4773	5342	2381
9	trans-α-Ocimene		LT 0.56^{NS}<br>Sit 3.02^{NS}<br>LT × Sit 0.04^{NS}	4784232	2005449	3790259	292998
10	α-Pinene		LT 1.04^{NS}<br>Sit 0.68^{NS}<br>LT × Sit 0.60^{NS}	548	104	50	36
11	α-Terpinene		LT 0.01^{NS}<br>Sit 5.21^{NS}<br>LT × Sit 6.02^{NS}	1116	1202	2418	22
12	α-Thujene		LT 3.83^{NS}<br>Sit 4.44^{NS}<br>LT × Sit 3.74^{NS}	1186	114	153	108

13	β-Myrcene		LT 2.54[NS]<br>Sit 0.15[NS]<br>LT × Sit 0.01[NS]	10189	8949	4114	2208
14	β-Ocimene		LT 0.54[NS]<br>Sit 3.61[NS]<br>LT × Sit 0.38[NS]	3309	978	2059	868
15	β-Phelladrene		LT 0.80[NS]<br>Sit 3.86[NS]<br>LT × Sit 0.60[NS]	1327	167	620	117
16	β-Pinene		**LT 7.30***<br>Sit 0.47[NS]<br>LT × Sit 1.73[NS]	92968	150052	53391	35502
17	γ-Terpinene		**LT 5.40***<br>Sit 0.19[NS]<br>LT × Sit 0.04[NS]	16341	13366	4610	3508
18	cis-Pinen-3-ol	monoterpene alcohol	LT 1.40[NS]<br>Sit 1.13[NS]<br>LT × Sit 1.46[NS]	882	51	7	60
19	Eucalyptol		LT 0.42[NS]<br>Sit 0.00[NS]<br>LT × Sit 1.52[NS]	124492	278959	194614	53771
20	Isoborneol		LT 1.48[NS]<br>Sit 4.42[NS]<br>LT × Sit 1.48[NS]	7041	156368	7160	46949
21	α-Terpineol		**LT 8.13***<br>**Sit 12.91****<br>**LT × Sit 9.04***	10220[b]	80003[a]	11854[b]	18054[b]
22	D-Germacrene	sesquiterpene	LT 0.11[NS]<br>Sit 3.47[NS]<br>**LT × Sit 4.22***	2554[a]	169[b]	1102[a]	1218[a]
23	α-Caryophyllene		LT 0.62[NS]<br>Sit 3.43[NS]<br>LT × Sit 0.00[NS]	43235[a]	19311[a]	33115[a]	8972[a]
24	α-Copaene		LT 0.62[NS]<br>**Sit 11.05***<br>LT × Sit 0.67[NS]	3517	601	2385	625
25	α-Cubebene		**LT 8.21***<br>**Sit 19.35****<br>LT × Sit 1.02	4460201	1371705	2247264	312465
26	α-Muurolene		**LT 9.49***<br>Sit 0.56[NS]<br>LT × Sit 0.99[NS]	14382	15236	7105	1038
27	β-Caryophyllene		LT 1.56[NS]	30708	18290	19500	12669

			Sit 2.04^{NS}<br>LT × Sit 0.17^{NS}				
28	β-Copaene		LT 2.46^{NS}<br>Sit 2.74^{NS}<br>LT × Sit 2.19^{NS}	1001	100	126	74
29	(z)-Hex-3-en-1-ol	alcohol	LT 0.06^{NS}<br>Sit 4.29^{NS}<br>LT × Sit 0.06^{NS}	2721741	7008	2159260	8617
30	1-Octen-3-ol		LT 0.05^{NS}<br>Sit 3.20^{NS}<br>LT × Sit 0.13^{NS}	22710	12017	23730	7653
31	2-Propenal	aldehyde	LT 0.89^{NS}<br>Sit 1.20^{NS}<br>LT × Sit 0.87^{NS}	900	94	153	88
32	Isovaleraldehyde		**LT 6.10***<br>Sit 0.52^{NS}<br>LTxSit 0.52^{NS}	81876770	46391341	3426789	3466464
33	Octenal		LT 2.94^{NS}<br>Sit 1.40^{NS}<br>LT × Sit 0.16^{NS}	10441	7566	6602	5171
34	α-Methyl-n-Butanal		LT 5.19^{NS}<br>Sit 0.00^{NS}<br>LT × Sit 0.02^{NS}	32740199	30174050	3454460	4492251
35	5-Methylheptan-3-one	keton	**LT 5.70***<br>Sit 0.21^{NS}<br>LT × Sit 0.08^{NS}	7776	8204	1578	3291
36	Pentan-3-one		**LT 7.73***<br>Sit 2.44^{NS}<br>LT × Sit 1.20^{NS}	321989	649080	114170	171753
37	β-tujone		**LT 17.37****<br>**Sit 6.21***<br>**LT × Sit 12.54****	65370^{b}	168599^{a}	54660^{b}	36692^{b}
38	(3z)-3-Hexenyl acetate	Aliphatic esters	LT 3.58NS<br>Sit 5.09NS<br>**LT × Sit 5.46***	1253^{a}	0^{b}	99^{ab}	122^{ab}
39	Dimethyl Sulfide	ether	**LT 23.77****<br>**Sit 5.34***<br>LT × Sit 1.40^{NS}	29866633	54386837	3926181	11857751

#Statistical analysis: two-way ANOVA with 4-9 replications (LT: leaf type; Sit: sites; LT × Sit: Leaf type × Sites interaction); *0.05 > p < 0.01; **0.01 > p < 0.001; ***0.001 > p; NS: not significant.

Table S2 – *F* statistic and *p* values (within brackets) of one-way ANOVA of the leaf-level photosynthetic parameters of *Salvia ceratophylloides* measured in 2016 and 2017.

Parameters	Statistics
$\mathbf{I_{comp}}$ [μmol(photon) m^{-2} s^{-1}]	0.406 (0.530)
$\mathbf{I_{max}}$ [μmol(photon) m^{-2} s^{-1}]	1.076 (0.311)
$\mathbf{I_{sat}}$ [μmol(photon) m^{-2} s^{-1}]	**5.263 (0.032)**
$\boldsymbol{P}_{\mathbf{N(Imax)}}$ [μmol(CO_2) m^{-2} s^{-1}]	3.115 (0.091)
$\boldsymbol{R}_{\mathbf{D}}$ [μmol(CO_2) m^{-2} s^{-1}]	3.808 (0.064)
$\phi_{(Icomp\text{-}200)}$ [μmol(CO_2) μmol(photon) $^{-1}$]	**4.561 (0.044)**
